# Quality of life vs. coping with stress by nurses during the COVID-19 pandemic

**DOI:** 10.3389/fpubh.2025.1664792

**Published:** 2025-10-01

**Authors:** Ewa Kupcewicz, Grażyna Piwko, Elżbieta Araminowicz-Kierklo, Katarzyna Młynarska-Antochów

**Affiliations:** ^1^Department of Nursing, Collegium Medicum, University of Warmia and Mazury in Olsztyn, Olsztyn, Poland; ^2^Branch in Elk, University of Warmia and Mazury in Olsztyn, Ełk, Poland

**Keywords:** quality of life, stress, coping, strategies, nurses

## Abstract

**Introduction:**

The COVID-19 pandemic, one of the greatest global health challenges of the 21st century, had a significant impact on the quality of life of healthcare personnel. It is par-ticularly nurses who, as front-line workers in the fight against the pandemic, have experi-enced a deterioration in their physical and psychosocial health. The increased workload, fear of infection, and stress associated with the epidemic situation had a significant impact on their well-being and quality of life.

**Aim of the study:**

To understand how nurses perceived their quality of life during the COVID-19 pandemic and to determine the role of stress-coping strategies in relation to the consequences of the spread of SARS-CoV-2 virus infections.

**Materials and methods:**

The study employed the diagnostic survey method with a group of 202 nurses working in hospitals in the city of Ełk between March and May 2021. The empir-ical data were collected using the WHOQoL-Bref Quality of Life Questionnaire, the Mini-COPE inventory, and a questionnaire developed by the authors of the study.

**Results:**

Significant correlations were observed between six stress-coping strategies and the psychological domain of the nurses’ quality of life. These include: “Active coping” (*r* = 0.242; *p* < 0.001), “Positive revalidation” (*r* = 0.153; *p* < 0.03), “Self-blaming” (*r* = −0.152; *p* < 0.03) and “Seeking instrumental support” (*r* = 0.227; *p* < 0.001). Analysis of predictors of quality of life showed that three stress-coping strategy groups, i.e., “A sense of humour,” “Helplessness” and “Seeking support,” achieved the R^2^ value in the regression model falling within the range of 0.02–0.08.

**Conclusion:**

Choosing appropriate stress-coping strategies can be an important factor in determining the nurses’ quality of life during crises such as a pandemic.

## 1 Introduction

Quality of life is a multidimensional concept whose definition varies depending on the research context and the field of science. The literature on the subject presents numerous approaches to defining quality of life. However, from a psychological perspective, the primary determinant of quality of life is the level of satisfaction an individual experiences in various aspects of their life existence. This satisfaction can reflect an overall assessment of one’s life or refer to its individual aspects, e.g., professional activity, family life, health status or social relationships (1, 2).

One of the most recognised definitions of quality of life has been proposed by the World Health Organization (WHO), defining it as “an individual’s perception of their position in life in the context of the culture and value systems in which they live, and in relation to their goals, expectations, standards and concerns” (3). According to this definition, quality of life covers all aspects of human functioning, which is the reason why the concept of quality of life-related to health is often used in medical sciences (Health-Related Quality of Life, HRQoL) (4, 5). This view emphasises the importance of physical and mental health as key determinants of an individual’s well-being.

The COVID-19 pandemic had a significant impact on nurses’ well-being, as documented in numerous scientific studies (6–11). Wasik and Koweszko demonstrated that the pandemic had an impact on mental health and sleep quality in nursing and midwifery personnel. It was found that 63% of respondents rated their sleep quality as low, and 35% exhibited signs of clinical insomnia. A stable mental state was positively correlated with the quality of sleep and negatively correlated with insomnia. In addition, 9% of the respondents received psychological help and showed lower scores in terms of mental health, poorer sleep quality, and higher levels of insomnia (6). A deterioration in the quality of sleep among nursing personnel was also documented in a study by Saracoglu et al. (7). Tomszczyszyn et al. found that 1/3 of nurses reported a low level of satisfaction with their lives (8). This level was influenced, e.g., by the place of residence and marital status, whereas previous infection with the SARS-CoV-2 virus had no significant effect on the nurses’ level of satisfaction with their lives (8). A study by Ginter et al. demonstrated a significant impact of the pandemic on female and male nurses’ mental well-being, leading to an increase in depressive symptoms and changes in the nature and characteristics of anxiety (9). The results of numerous scientific studies conducted in recent years indicate the adverse impact of the COVID-19 pandemic on the health of medical personnel, especially in the context of anxiety, depression, sleep disorders, increased stress and professional burnout (10–14). Many healthcare professionals also suffered from compassion fatigue (15, 16).

Due to the specific nature of the nursing profession, the issue of coping with stress and its impact on quality of life is a key topic in the field of health psychology. Modern theoretical stress models are primarily based on the transactional approach that defines stress as a dynamic interaction between an individual and their environment. Recent research into stress-coping mechanisms indicates a shift in perspective from the dominance of cognitive processes to the emotional aspect, with emotions being perceived as an integral part of the stress transaction (17). This shift has significant implications for both the theory of stress and the strategies of psychological interventions aimed at healthcare professionals.

From the onset of the COVID-19 pandemic, healthcare personnel worldwide experienced a significant psychophysical burden resulting from excessive hours, exposure to stressors, and the risk of infection (18). In response to these challenges, a variety of stress-coping strategies were employed, both adaptive and non-adaptive. A study by Lemska et al. showed that the most common coping strategy used by both female and male nurses during the COVID-19 pandemic was “Active coping.” Furthermore, the frequency of this coping style was positively correlated with the age of the respondents (19). In turn, Ferreira et al. demonstrated that a positive attitude was a strong and significant predictor of well-being and mental adaptation during the pandemic crisis. Of the stress-coping strategies used by healthcare personnel, “Denial,” “Self-blaming,” and “Taking care of something else” were associated with poorer adaptation and more severe deterioration of mental health. “Self-blaming” appeared to be the most damaging strategy (20).

In view of the long-term effects of the pandemic, it was considered crucial to research its impact on healthcare professionals’ quality of life, which can form the basis for the development of effective psychological and institutional support strategies.

The study aimed to understand how nurses perceived their quality of life during the COVID-19 pandemic and to determine the role of stress-coping strategies in relation to the consequences of the spread of SARS-CoV-2 virus infections.

To achieve the objective, the following research questions were asked:

1. Are there differences in the quality of life and stress-coping strategies among nurses working in two hospitals in the city of Ełk, and if so, how significant are they?
2. Are there any correlations between the stress-coping strategies and the nurses’ quality of life, and if so, how strong are they? Which stress-coping strategies can be used as predictors of the quality of life in the group of nurses under study?

## 2 Materials and methods

### 2.1 Settings and design

The study was conducted using the diagnostic survey method in a group of 202 nurses employed in hospitals in the city of Ełk between March and May 2021. After being granted permission to conduct the study by the hospital management, the researchers personally delivered the survey questionnaires to the facilities. The study was conducted while respecting the sanitary regime rules. The nurses participating in the study were informed of the aim of the study and had the opportunity to ask questions and receive comprehensive information. Once the nurses agreed to participate in the study, 220 questionnaire packages were distributed. The survey took approximately 15 min to complete. Participation in the survey was voluntary, and the nurses could opt out of the study at any time without giving a reason. After collecting the data and eliminating incomplete questionnaires, 202 questionnaires (a response rate of 91.82%) were included for further statistical analysis, including 102 from nurses at the 1st Military Clinical Hospital with Independent Local Outpatient Clinic in Lublin, Branch in Ełk (subgroup 1), and 100 from nurses at the “Pro-Medica” Municipal Hospital with the CK Scanmed Cardiology Department in Ełk (subgroup 2). The study was conducted in accordance with the Declaration of Helsinki and the positive opinion obtained from the Senate Research Ethics Committee of the Józef Rusiecki Higher School in Olsztyn, Poland (No 11/2016).

### 2.2 Research instruments

The empirical data were collected using the following:

- A self-designed questionnaire which contained questions about socio-demographic details, including age, sex, marital status, place of residence, job position, years of service, material status and the number of children;
- The Quality of Life Questionnaire—a WHOQoL-Bref version *adapted to Polish conditions by L. Wołowicka and K. Jaracz* (21);
- The Mini-COPE inventory *by C. S. Carver adapted to Polish conditions* (17).

#### 2.2.1 The WHO-bref questionnaire

The WHOQoL-Bref questionnaire is an abridged version of the WHOQoL-100, developed by a team of researchers at the World Health Organization (WHO) and designed to assess the quality of life of healthy and diseased individuals. It contains 28 questions, of which 26 enable the respondent to obtain a quality of life profile in the physical (somatic) health domain, psychological domain, social relations domain and community domain. The other two questions, analysed separately, concern the individual’s overall perception of their quality of life and health. Respondents provide answers on a 5-point Likert scale (the scoring range is from 1 to 5, where 1 - very dissatisfied, and 5 = very satisfied). In each domain, the respondent can score a maximum of 20 points. The scale for the results for the individual domains is a positive score (meaning that the higher the score, the better the quality of life). The questionnaire has good psychometric properties (21).

#### 2.2.2 Inventory to measure coping with stress—mini-COPE

The Mini-COPE inventory is a self-report tool designed to measure dispositional coping, i.e., to assess typical ways of reacting and feeling when experiencing high levels of stress. The inventory contains a total of 28 statements that make up 14 stress-coping strategies (two statements for each strategy). For each statement, the respondent marks one of the four possible answers that indicate the following: 0 – I almost never do this; 1 – I rarely do this; 2 – I often do this; 3 – I almost always do this. The results were either analysed separately for each strategy or grouped according to the common features of the factorial scale structure. Seven stress-coping strategy groups were distinguished: “Active coping,” “Helplessness,” “Seeking support,” “Avoidance,” “A turn to religion,” “Acceptance,” and “A sense of humour.” The psychometric indicators of the original version of the Mini-COPE inventory are considered good (Cronbach’s *α* = 0.70) (17).

### 2.3 Statistical analysis

The data generated during the *a posteriori* study were subjected to statistical analysis using the Polish version of STATISTICA 13 (TIBCO, Palo Alto, CA, United States). Socio-demographic data is presented as the number of cases and the % value. For the description of the analysed variables, the following were used: mean value, standard deviation, median, confidence interval for the mean value of ±95%, minimum and maximum. In order to examine the significance of differences in the stress-coping strategies used and the WHOQoL-Bref quality of life indicators, an analysis of variance ANOVA (F) test was used to compare multiple samples of independent groups. In turn, Pearson’s correlation (r) was used to examine the significance of the strength and direction of the relationship between the stress-coping strategy indicators and the quality of life. The search for predictors of quality of life used multiple regression analysis (22). A significance level of *p < 0.05* was adopted for all tests.

## 3 Results

### 3.1 Participants

A total of 202 nurses participated in the study, including 193 women (95.54%) and nine men (4.46%). The most numerous group consisted of individuals aged between 46 and 55 (*n* = 90; 44.55%). More than 70% (*n* = 145) of the respondents reported that they worked in (day/night) shifts. Of the respondents, 30.69% had worked in the profession for more than 30 years. More than half (*n* = 107; 52.97%) of the participants stated that their material status was good. Detailed data is provided in Table 1.

**Table 1:** Characteristics of the respondents.

Variables		Total *N* = 202
		n (%)
Type of job position	Managerial	26 (12.87)
	Executive	176 (87.13)
System of work	Single-shift	57 (28.22)
	Shift	145 (71.78)
Sex	Woman	193 (95.54)
	Man	9 (4.46)
Age (in years)	25–35	30 (14.85)
	36–45	43 (21.29)
	46–55	90 (44.55)
	56 and over	39 (19.31)
Marital status	In a relationship	150 (74.26)
	Single	52 (25.74)
Place of residence	Village	45 (22.28)
	City population up to 50,000	34 (16.83)
	Population over 50,000	123 (60.89)
Years of service	1–10	36 (17.82)
	11–20	50 (24.75)
	21–30	54 (26.73)
	31 and more	62 (30.70)
Number of years in the last position held	1–10	85 (42.07)
	11–20	53 (26.24)
	21–30	37 (18.32)
	31 and more	27 (13.37)
Holding a disability certificate	Yes	8 (3.96)
	No	194 (96.04)
Material status	Very good	16 (7.92)
	Good	107 (52.97)
	Satisfactory	65 (32.18)
	Poor	14 (6.93)

### 3.2 Differences in the quality of life of the nurses under study

Statistical analyses were used to evaluate the differences in WHOQoL-Bref scores and stress-coping strategies among the nurses under study, taking into account the workplace (subgroup 1; subgroup 2). An analysis of variance (ANOVA) test did not show any statistically significant differences in the mean values in all domains of the nurses’ quality of life, regardless of the hospital where the nurses worked (Table 2).

**Table 2:** Differences in the quality of life of the nurses under study.

Item	Variables WHOQoL-Bref	Subgroup 1 *n* = 102	Subgroup 2 *n* = 100	ANOVA test (F)	*p*-value
		M ± SD, Me, Min. – Max., −95, +95%	M ± SD, Me, Min. – Max., −95, +95%		
1.	Q1	3.77 ± 0.70, 4 1–5 3.64–3.91	3.87 ± 0.54, 4 3–5 3.76–3.98	1.16	0.282
2.	Q2	3.62 ± 0.86, 4 1–5 3.45–3.79	3.65 ± 0.67, 4 1–5 3.52–3.78	0.09	0.766
3.	D1	12.90 ± 1.95, 13 9–18 12.51 = −13.28–5	12,52 ± 1.82, 12 7–18 12.16–12.88	2.01	0.158
4.	D2	14.42 ± 1.74, 15 11–19 14.08–14.76	14.09 ± 1.71, 14 9–18 13.75–14.43	1.80	0.182
5.	D3	1.28 ± 2.66, 16 9–20 14.76–15.80	15.00 ± 2.53, 16 8–20 14.50–15.50	0.59	0.443
6.	D4	13.82 ± 2.13, 14 8–18 13.41–14.24	13.90 ± 2.20, 14 7–20 13.46–14.34	0.06	0.802

Statistically significant: *p* < 0.05; *p* < 0.01; *p* < 0.001. Explanation: Q1, satisfaction with overall quality of life; Q2, satisfaction with overall health quality; D1, somatic domain; D2, psychological domain; D3, social domain; D4, community domain; M, mean; SD, standard deviation; Me, median; Min., minimum; Max., maximum; confidence interval for the mean value of ±95%.

### 3.3 Differences in the stress-coping strategies used in the group under study

Subsequent statistical analyses using the ANOVA variance analysis test showed significant differences in the mean values for the strategy of “Planning” (*F* = 6.44; *p* < 0.01) in favour of the respondents from subgroup 2. In contrast, nurses in subgroup 1 achieved significantly higher mean values for the following strategies: “The use of psychoactive substances” (*F* = 7.76; *p* < 0.006), “Cessation of actions” (*F* = 7.42; *p* < 0.007), “Denial” (*F* = 18.63; *p* < 0.0002), and “A sense of humour” (*F* = 12.63; *p* < 0.0005; Table 3).

**Table 3:** Differences in the stress-coping strategies used in the group under study.

Item	Variables (Mini-Cope)		Subgroup 1 *n* = 102	Subgroup 2 *n* = 100	ANOVA test (F)	*p*-value
			M ± SD, Me, Min. – Max., −95, +95%	M ± SD, Me, Min. – Max., −95, +95%		
1.	Active coping	Active coping	2.04 ± 0.61, 2 0–3 1.92–2.16	2.14 ± 0.61. 2 1–3 2.02–1.26	1.30	0.25
		Planning	1. ± 0.71, 2 0–3 1.77–2.05	2.15 ± 0.61. 2 0–3 2.03–1.26	6.44	0.01
		Positive revalidation	1.83 ± 0.66, 2 0–3 1.70–1.96	1.84 ± 0.61. 2 0–3 1.71–1.97	0.01	0.94
2.	Helplessness	Use of psychoactive substances	0.42 ± 0.67, 0 0–3 0.29–0.55	0.20 ± 0.61. 2 0–2 0.12–0.28	7.76	0.006
		Cessation of actions	1.03 ± 0.69. 1 0–3 0.90–1.16	0.78 ± 0.61. 2 0–3 0.65–0.90	7.42	0.007
		Self-blaming	1.19 ± 0.72, 1 0–3 1.05–1.33	1.05 ± 0.61. 2 0–3 0.91–1.18	2.27	0.13
3.	Seeking support	Seeking emotional support	1.82 ± 0.73,2 0–3 1.68–1.97	1.87 ± 0.61. 2 0–3 1.71–2.02	0.15	0.69
		Seeking instrumental support	1.75 ± 0.61, 2 0–3 1.61–1.88	1.69 ± 0.61. 2 0–3 1.54–1.84	0.29	0.58
4.	Avoidance	Taking care of something else	1.83 ± 0.63, 2 0–3 1.71–1.95	1.67 ± 0.61. 2 0–3 1.52–1.81	2.96	0.08
		Denial	1.16 ± 0.67, 1 0–3 1.02–1.29	0.75 ± 0.61. 2 0–3 0.62–0.88	18.63	0.0002
		Discharge	1.35 ± 0.64, 2 0–3 1.23–1.48	1.19 ± 0.61. 2 0–3 1.05–1.32	3.33	0.070
5.	A turn to religion		1.25 ± 0.96, 1 0–3 1.06–1.43	1.49 ± 0.61. 2 0–3 1.32–1.66	3.70	0.056
6.	Acceptance		1.77 ± 0.58, 2 0–3 1.66–1.89	1.93 ± 0.61. 2 1–3 1.82–2.04	3.63	0.058
7.	A sense of humour		1.02 ± 0.64, 1 0–3 0.90–1.15	0.70 ± 0.61. 2 0–3 0.57–0.83	12.63	0.0005

Statistically significant: *p* < 0.05; *p* < 0.01; *p* < 0.001. Explanation: M, mean; SD, standard deviation; Me, median; Min., minimum; Max., maximum; confidence interval for the mean value of ±95%.

### 3.4 The nature and degree of intensity of the relationships between the preferred stress-coping strategies and the respondents’ quality of life

The study used the r-Pearson correlation coefficient to determine the nature and degree of intensity of the relationships between the preferred stress-coping strategies and the nurses’ quality of life. The results obtained (Table 4) indicated that only some of the stress-coping strategies show significant correlations with the individual domains of quality of life during the COVID-19 pandemic. Particular attention was paid to significant correlations between six stress-coping strategies and the psychological domain of the nurses’ quality of life. Among these strategies, the following stand out: “Active coping” (*r* = 0.242; *p* < 0.001), “Positive revalidation” (*r* = 0.153; *p* < 0.03), “Self-blaming” (*r* = −0.152; *p* < 0.03) and “Seeking instrumental support” (*r* = 0.227; *p* < 0.001), all of them having a significant impact on the nurses’ mental health during the pandemic.

**Table 4:** The nature and degree of intensity of the relationships between the preferred stress-coping strategies and the respondents’ quality of life—r-Pearson’s correlation coefficients; *N* = 202.

Item	Variables (Mini-Cope)		WHOQoL-Bref											
			Q1		Q2		D1		D2		D3		D4	
			r	*p*	r	*p*	r	*p*	r	*p*	r	*p*	r	*p*
1.	Active coping	Active coping	0.083	0.23	0.035	0.64	0.039	0.58	0.242	0.001	0.113	0.11	0.068	0.33
		Planning	0.071	0.31	0.044	0.58	−0.003	0.96	0.036	0.61	0.079	0.26	0.041	0.56
		Positive revalidation	0.116	0.1	0.053	0.45	0.165	0.01	0.153	0.03	0.087	0.22	0.157	0.03
2.	Helplessness	Use of psychoactive substances	−0.045	0.52	0.018	0.8	−0.035	0.62	−0.113	0.1	−0.122	0.08	−0.077	0.27
		Cessation of actions	−0.129	0.06	−0.140	0.04	−0.067	0.34	−0.091	0.19	−0.188	0.007	−0.181	0.01
		Self-blaming	−0.128	0.06	−0.110	0.12	−0.091	0.19	−0.152	0.03	−0.137	0.06	−0.214	0.002
3.	Seeking support	Seeking emotional support	0.146	0.03	0.030	0.67	0.081	0.25	0.192	0.006	0.251	0.0001	0.201	0.004
		Seeking instrumental support	0.070	0.32	−0.017	0.81	0.121	0.08	0.227	0.001	0.267	0.0001	0.157	0.03
4.	Avoidance	Taking care of something else	−0.001	0.98	−0.026	0.71	0.051	0.47	0.097	0.17	−0.001	0.96	−0.032	0.64
		Denial	−0.012	0.86	−0.054	0.44	0.039	0.5	−0.030	0.66	−0.063	0.37	−0.086	0.22
		Discharge	−0.124	0.08	−0.089	0.2	−0.020	0.77	−0.040	0.57	0.004	0.950	−0.084	0.23
5.	A turn to religion		−0.029	0.68	−0.038	0.58	0.102	0.15	0.072	0.31	0.033	0.64	0.033	0.63
6.	Acceptance		0.077	0.27	0.072	0.3	−0.031	0.65	0.046	0.51	0.014	0.84	0.041	0.56
7.	A sense of humour		0.025	0.72	0.014	0.84	0.202	0.004	0.251	0.0001	0.135	0.055	0.075	0.29

Statistically significant: *p* 0.05; *p* < 0.01; *p* < 0.001. Explanation: Pearson’s r, Q1, satisfaction with overall quality of life; Q2, satisfaction with overall health quality; D1, somatic domain; D2, psychological domain; D3, social domain; D4, community domain.

### 3.5 Predictors of nurses’ quality of life

An attempt was made to determine the predictors of nurses’ quality of life during the COVID-19 pandemic. Of the dependent variables, four domains of quality of life were considered, including somatic, psychological, social and community. In contrast, the explanatory variables included the following stress-coping strategy groups: “Active coping,” “Helplessness,” “Seeking support,” “Avoidance,” “A turn to religion,” “Acceptance,” and “A sense of humour.” Analysis of the results provided in Table 5 showed that only some of the stress-coping strategies were included in the regression model. The strategies of “A sense of humour” and “Helplessness” turned out to be significant predictors of quality of life in all the nurses’ functioning domains under study.

**Table 5:** Summary of regression—nurses’ quality of life during the COVID-19 pandemic.

Variables			R^2^	*ßeta*	ß	ß-error	t	*p*-value
WHOQoL-Bref	D1	A sense of humour	0.04	0.28	0.78	0.21	3.75	0.00
		Helplessness	0.03	−0.21	−0.85	0.30	−2.83	0.01
		R = 0.29; R^2^ = 0.07; corrected R^2^ = 0.06						
	D2	A sense of humour	0.06	0.34	0.88	0.18	4.87	0.00
		Helplessness	0.08	−0.31	−1.14	0.26	−4.42	0.00
		Seeking support	0.04	0.20	0.52	0.17	3.10	0.00
		R = 0.42; R^2^ = 0.18; corrected R^2^ = 0.17						
	D3	Seeking support	0.08	0.33	1.29	0.29	4.46	0.00
		Helplessness	0.05	−0.27	−1.49	0.44	−3.42	0.00
		A sense of humour	0.04	0.23	0.89	0.28	3.16	0.00
		R = 0.42; R^2^ = 0.18; corrected R^2^ = 0.16						
	D4	Helplessness	0.05	−0.25	−1.19	0.37	−3.17	0.00
		Seeking support	0.04	0.22	0.71	0.23	3.09	0.00
		A sense of humour	0.02	0.18	0.60	0.24	2.46	0.01
		R = 0.35; R^2^ = 0.12; corrected R^2^ = 0.10						

Statistically significant: *p* < 0.05; *p* < 0.01; *p* < 0.001. R, correlation coefficient; R^2^, multiple determination coefficient; ßeta, standardised regression coefficient; B, non-standardised regression coefficient; Error B, non-standardised regression coefficient error; t, t-test value.

The regression model obtained for the somatic domain explained 7% of the variability of the results. The regression coefficient for the strategy of “A sense of humour” as a predictor took on a positive value (*β* = 0.28; R^2^ = 0.04), which indicates a positive relationship. This result suggests that “A sense of humour” may function as a mechanism to alleviate negative emotions among nurses. In turn, the strategy of “Helplessness” explained 3% of the variability of the results and took on a negative value (*β* = −0.21; R^2^ = 0.03), which may indicate its adverse effect on the quality of life in this particular domain.

Analysis of predictors of quality of life in the psychological domain showed that three variables were included in the regression model, which together explained 18% of the variability of the results. The strategy of “A sense of humour” was responsible for 6% of the variability, “Helplessness” for 8%, whereas the strategy of “Seeking support” was responsible for 4%.

A similar pattern was observed in the social domain, where the same three variables together explained 18% of the variability of the results. In this domain, the strategy of “Seeking support” was responsible for 8% of the variability, “Helplessness” for 5%, whereas the strategy of “A sense of humour” was responsible for 4%.

In summary, the regression model achieved the R^2^ value ranging from 0.02 to 0.08, meaning that it only explains a small part of the variability of the dependent variable.

## 4 Discussion

The present study attempted to understand how nurses employed in hospitals in the city of Ełk perceived their quality of life during the COVID-19 pandemic and determine the role of stress-coping strategies in the context of the consequences of the spread of SARS-CoV-2 virus infections. A review of the available literature indicates that nurses perceive stress as an integral part of their profession. In the face of a threat, they take active measures aimed at reducing it by planning their work and maintaining a positive attitude (23). Nurses, facing the challenges associated with the COVID-19 pandemic and providing direct care to patients infected with the SARS-CoV-2 virus, experienced unprecedented mental pressure due to their heavy workload, the risk of infection, and the need to make quick decisions under conditions of limited resources (24–26).

The authors’ own study noted no significant differences in the level of nurses’ quality of life, depending on their workplace. In the regression models, three groups of stress-coping strategies—sense of humor, helplessness, and seeking social support—emerged as the primary predictors of quality of life across all functional domains. Collectively, these variables accounted for up to 18% of the variance in outcomes. Given that quality of life is a complex, multifactorial construct shaped by a broad range of influences, this level of predictive power was considered acceptable, particularly as the variables included in the models captured only a limited portion of its determinants.

The strategy of “A sense of humour” may have played an important role in minimising the adverse effect of occupational stress, thus enabling nurses to cope more efficiently with difficult situations. At the same time, its use could indicate a strategy of distancing from problems instead of solving them directly. The results of the study confirmed that social support played a crucial role in the daily functioning of nurses in the face of hazards to health and life. They sought advice, assistance, information as well as positive feelings and understanding from their colleagues and relatives. Their coping strategies focused on both the problem and on regulating their emotions, which indicates their adaptive approach to work-related challenges.

As demonstrated by Gniadek et al., nurses actively participated in mutual support groups during the COVID-19 pandemic. They consciously exchanged scientific articles, discussed guidelines and shared their observations, which facilitated the exchange of experiences and the improvement of professional practice (23). The authors emphasise that professionalism, high professional competence and communication skills at the highest level are crucial qualities that every person providing medical care to a patient should possess (23). There is significant scientific evidence confirming that social support played a crucial role in maintaining the mental health of healthcare professionals during the COVID-19 pandemic (27). A study by Xu et al. demonstrated that social support may act as a mediator in the process of coping with stress, strengthen mental resilience, and positively influence the mental health of medical personnel (28). A study conducted by Burba and Gotlib showed that 84% of nurses consider stress to be an integral part of their job. The most burdensome factors included time pressure and responsibility for patients’ health and lives. Therefore, nurses use various stress-coping strategies, including engaging in certain health-related behaviours aimed at reducing tension (29). In the same study by Burba and Gotlib, 65% of the respondents admitted that they reached for cigarettes to alleviate stress (29). A study conducted by Szlenk-Czyczerska et al. during the COVID-19 pandemic in both public and non-public healthcare facilities in Opolskie and Lubelskie voivodeships found that nurses coped with stress mainly through the strategy of “Active coping,” including “Planning,” “Acceptance,” “Positive revalidation” and “Seeking instrumental support.” These stress-reducing mechanisms allowed them to maintain their psychophysical balance during the difficult period of the pandemic (30). In turn, Bluszcz and Matachowska, when analysing the consequences of long-term increased workload affecting nurses during the pandemic, showed that the nurses under study did not report any increasing mental problems. Instead, they focused on early self-prevention strategies aimed at preventing uncontrolled deterioration of psychophysical conditions. Thanks to these interventions, despite the ongoing pandemic, they were able to maintain a relatively good state of well-being and derive satisfaction from performing their work, even in the face of difficult and stressful situations (31). It is worth noting, however, that the results of other studies are less optimistic. One example is a study conducted on a group of 14,825 physicians and nurses in 31 provinces of China during the first year of the COVID-19 pandemic. The study evaluated the health status of the medical personnel working in Accident and Emergency Departments and found that the prevalence of depression and post-traumatic stress disorder (PTSD) symptoms was 25.2 and 9.1%, respectively. In addition, being a nurse was associated with a higher risk of developing PTSD, as compared to other occupational groups (32).

De Kock et al. performed a literature review based on an analysis of 24 studies, with 18 of them conducted in China, and demonstrated that the COVID-19 pandemic had a significant impact on the mental well-being of healthcare professionals, especially those working on the so-called ‘frontline’. The researchers indicated that nurses were particularly exposed to an increased risk of negative effects on their mental health in the course of their professional duties (33). Numerous studies on the restrictions imposed during the COVID-19 pandemic highlight that social isolation, which was enforced for epidemiological reasons had a significant impact on the psychosocial health and quality of life of individuals across various age groups (34, 35). In the authors’ own study, the nurses gave the highest rating to the quality of life in the area of functioning in social relationships, whereas the lowest rating to the quality of life in the area of physical health. Many researchers note that nurses working on the frontline of patient care during the COVID-19 pandemic were at greater risk of isolation, discrimination, and increased susceptibility to physical fatigue, emotional disturbances, and sleep problems (36–39).

These findings are consistent with those of Reynolds et al., who highlighted that in the health care system of the United States of America, the COVID-19 pandemic exposed the critical role of the nursing subsystem in ensuring continuity and quality of care (40). Insufficient staffing levels, excessive workload, and the deterioration of nurses’ mental health significantly limited the system’s ability to respond effectively to the crisis. This underscores the need for strategic strengthening of nursing resources as a fundamental component of health system resilience (40).

The quality of life of nurses during the COVID-19 pandemic was associated with multiple factors. Findings from the study by Senosy, conducted among nurses in Egypt, indicate that the level of knowledge, practical skills, and professional experience influenced work performance, while environmental factors—such as the availability of resources and team support—could help reduce stress burden in crisis situations (41). Similar observations were reported by Fernandez et al. (42) and Zhang et al. (43), who emphasised the importance of ensuring appropriate working conditions, including access to training and sufficient resources, to improve both the quality of performed duties and the psychological well-being of nurses.

## 5 Limitations and implications for professional practice

The present study has certain limitations, with one major issue being the lack of questions in the questionnaire regarding the participants’ quality of life before the introduction of COVID-19-related restrictions. Such data could enable a more detailed analysis of the impact of the pandemic on nurses’ mental and physical health, enabling a comparison of the results to the pre-pandemic health status. In addition, the study did not take into account the long-term mental effects that could arise after the health crisis has ended. Another limitation of the study is the relatively low explanatory power of the regression models (R^2^ ranging from 0.02 to 0.18). This indicates that the coping strategies included in the analysis explain only a limited proportion of the variance in nurses’ quality of life, while other important predictors—such as workload, financial situation, or individual resilience—were not included in this study. Future research should therefore incorporate a broader range of variables to provide a more comprehensive understanding of the factors influencing nurses’ quality of life. From the perspective of professional practice, the study results indicate the need to develop institutional psychological support and implement stress reduction programmes that could quickly and effectively help relieve the tension associated with the demands of working under crisis conditions. Moreover, it is advisable to implement social support systems for medical personnel to prevent isolation and discrimination and create conditions conducive to better management of the mental and physical health of medical personnel. The implementation of such solutions could contribute to maintaining the high quality of nursing care and improving the psychophysical condition of nurses, which is particularly important in the context of future health crises. The proposed strategic solutions are also supported by the recommendations of other researchers (40–44).

## 6 Conclusion

1. The study did not observe any differences in the quality of life among nurses working in two hospitals in Ełk. However, certain differences were found in the strategies the nurses used to cope with stress, including “Planning,” “The use of psychoactive substances,” “Cessation of actions,” “Denial,” and “A sense of humour.”
2. The study confirms that choosing appropriate stress-coping strategies can be an important factor determining the nurses’ quality of life during crises such as a pandemic. The results suggest that the strategies of “Active coping,” “Seeking support,” and “A sense of humour” can contribute to better maintenance of mental health among healthcare personnel, which, in turn, contributes to an improvement in their well-being and effectiveness in performing their professional duties.
3. Three stress-coping strategy groups, namely “A sense of humour,” “Helplessness,” and “Seeking support,” were demonstrated to be significant predictors of the quality of life of the nurses under study.
4. There is a need to promote active stress-coping strategies among nurses and other healthcare professionals in order to support their mental and physical well-being and ensure the high quality of the care provided.

## Data Availability

The original contributions presented in the study are included in the article/supplementary material, further inquiries can be directed to the corresponding author.
